# Quality in clinical cytometry

**DOI:** 10.1186/1476-9255-12-S1-O5

**Published:** 2015-04-16

**Authors:** David WL Wilson

**Affiliations:** 1 Department of Pathology, Aberdeen Royal Infirmary, Aberdeen, AB25 2ZD, UK

## 

To understand how we achieve quality within a clinical flow cytometry setting we need to appreciate the difference between Quality Control (QC) and Quality Assurance (QA). QC may be defined as a procedure or set of procedures intended to ensure that a manufactured product or performed service adheres to a defined set of quality criteria. In contrast QA is the planned and systematic activities implemented in a quality system so that quality requirements for a product or service will be fulfilled. In general, one can look at QA as the overall description and control of a process while QC plays a part in control of the process. When looking at QA we need to consider how we control the pre analytical, analytical and post analytical process. These processes need to be documented and training and competence evidenced. The pre analytical process includes defining the specimen type that is acceptable, how it will be transported, what identification is required and whether this is the correct test for the patient. The analytical process is the part of the process that most scientists are familiar with. We have to ensure that we conduct the test using the correct methodology using suitable equipment (validation) and the reagents are suitable for purpose (verification). As part of the evidence for validation it is normal to refer to reference methods, published guidelines, biological reference material, external and internal QC. The post analytical process, like in the pre analytical process, is where we again have to look at how the laboratory interfaces with it’s users. Consideration has to be given to who is competent to interpret reports and advise service users, how we are going to report laboratory results so that the user understands them and finds them an aid to diagnosis and treatment. The International Organization for Standardization has developed a set of standards for medical laboratories, ISO 15189:2012. In the United Kingdom these standards have been adopted to replace the Clinical Pathology Accreditation standards and are accredited by the United Kingdom Accreditation Service (UKAS). The ISO 15189:2012 standards require laboratories to pay more attention to traceability of methods through the use of reference material (WHO, NIBSC etc.), reference methods and calibration as well as the competence, qualification and training of laboratory staff, how this is assessed and reviewed. Through adoption of these processes clinical flow cytometry laboratories can demonstrate robust analysis to support clinical diagnosis and are equipped to deal with the changing environment of clinical flow cytometry.

